# The relationship between childhood trauma and non-suicidal self-injury in adolescents with depressive disorders: the mediating role of alexithymia and coping strategies

**DOI:** 10.3389/fpsyt.2026.1744863

**Published:** 2026-03-13

**Authors:** Yindu Liu, Yihui Liu, Rui Zhou, Cailan Hou

**Affiliations:** Guangdong Mental Health Center, Guangdong Provincial People’s Hospital (Guangdong Academy of Medical Sciences), Southern Medical University, Guangzhou, Guangdong, China

**Keywords:** childhood trauma, non-suicidal self-injury, alexithymia, coping, adolescents, depression

## Abstract

**Background:**

Non-suicidal self-injury (NSSI) is highly prevalent among adolescents with depressive disorders and is associated with adverse clinical outcomes. Childhood trauma is a well-established risk factor for NSSI, yet the psychological mechanisms underlying this association remain unclear. This study examined the mediating roles of alexithymia and coping strategies in the relationship between childhood trauma and NSSI among adolescents with depressive disorders.

**Methods:**

A total of 129 patients (aged 12–18 years, mean age = 14.74) with depressive disorders and non-suicidal self-injury (NSSI) were recruited from the inpatient and outpatient departments of the hospital. Diagnostic interviews were conducted to diagnose depressive disorders and NSSI by professional psychiatrists. Demographic characteristics of those depressed adolescents were collected using a self- administered questionnaire. The Childhood Trauma Questionnaire Short Form (CTQ-SF), the Adolescent Non-suicidal Self‐injury Assessment Questionnaire (ANSAQ), the Chinese version of the 20-item Toronto Alexithymia Scale (TAS-20-C) and the Simplified Coping Style Questionnaire (SCSQ) were used to obtain information about childhood trauma experience, NSSI, alexithymia and coping.

**Results:**

Emotional neglect and emotional abuse were the most prevalent trauma subtypes. Childhood trauma was positively correlated with NSSI (r = 0.599, p < 0.001), alexithymia (r = 0.525, p < 0.001), and negative coping (r = 0.338, p < 0.001), and negatively correlated with positive coping (r = –0.392, p < 0.001). Mediation analyses showed that alexithymia (β = 0.075, 17.08%) and negative coping (β = 0.032, 7.29%) partially mediated the relationship between childhood trauma and NSSI. Moreover, alexithymia and negative coping formed a significant chain mediation pathway (β = 0.028, 6.38%).

**Conclusions:**

Childhood trauma contributes to NSSI both directly and indirectly via alexithymia and maladaptive coping. Targeted interventions addressing emotional awareness and coping flexibility may help reduce the risk of self-injury among adolescents with depressive disorders and trauma histories.

## 1 Introduction

Non-suicidal self-injury (NSSI) is a prevalent and serious mental health concern among adolescents and young adults, characterized by the deliberate destruction of body tissue without suicidal intent (1–4). The prevalence of NSSI can reach as high as 51% among hospitalized adolescents with psychiatric disorders (5). In clinical settings, NSSI is associated with prolonged hospitalization, poorer prognosis, and an increased risk of suicide (6). Given these clinical consequences, identifying the underlying risk factors and early psychological mechanisms of NSSI is critical for developing effective prevention and intervention strategies.

Several well-established, textbook-level theoretical frameworks provide an essential foundation for understanding the development and maintenance of NSSI. Firstly, Nock’s Four-Function Model remains the most widely recognized explanation for why NSSI behaviors are maintained. The Four-Function Model posits that NSSI serves four reinforcement functions: automatic negative reinforcement, automatic positive reinforcement, social negative reinforcement, and social positive reinforcement (7). This model highlights emotion regulation as a core intrapersonal function of NSSI and provides a foundational explanation for why self-injurious behaviors persist once established. While this model offers a parsimonious explanation for why self-injurious behaviors persist once established, it is less explicit in accounting for why certain individuals are more vulnerable to developing NSSI.

Developmental trauma theories (8) address this vulnerability by emphasizing that exposure to chronic or interpersonal trauma during childhood disrupts normative emotional and self-related development, thereby conferring long-term risk for maladaptive coping and psychopathology. Within this broader theoretical tradition, the Constructivist Self-Development Theory (CSDT) provides a specific psychological account of how childhood trauma impairs core self-capacities, including emotional awareness, affect regulation, and identity coherence. Such trauma-related disruptions are thought to predispose individuals to difficulties in managing internal distress (9). Complementing this perspective, the experiential avoidance model (10) conceptualizes NSSI as an extreme behavioral attempt to escape, suppress, or modulate overwhelming internal experiences, thereby linking emotional processing deficits to self-injurious behavior. Taken together, these frameworks converge on the view that childhood trauma functions as a distal vulnerability factor that increases the risk of NSSI through disruptions in emotional processing and self-regulation.

Consistent with these theoretical perspectives, empirical research has robustly linked childhood trauma—particularly emotional abuse and emotional neglect—to NSSI (11, 12). However, trauma exposure alone is insufficient to explain self-injurious behavior; rather, its effects are largely mediated by impairments in emotional processing and regulation. Alexithymia, characterized by difficulties in identifying and describing one’s own emotions, represents a core manifestation of such impairments (12). From a developmental trauma perspective, alexithymia reflects compromised emotional self-capacities resulting from trauma-related disruptions in affect integration (13). Adolescents with elevated alexithymia may experience intense negative affect without adequate emotional insight, increasing their reliance on maladaptive behavioral strategies to achieve emotional relief.

Coping strategies constitute the behavioral expression of emotional regulation capacities and represent a key pathway through which emotional processing deficits may translate into self-injurious behavior. Adaptive coping strategies, such as problem solving and cognitive reappraisal, are generally associated with better psychological adjustment (14), whereas maladaptive or negative coping strategies—including avoidance, withdrawal, and self-blame—are more prevalent among individuals with trauma histories and emotional processing deficits (15). Importantly, alexithymia may constrain the effective use of adaptive coping strategies, thereby increasing reliance on maladaptive coping patterns that align with experiential avoidance and the intrapersonal negative reinforcement function of NSSI (13, 14). This suggests a theoretically ordered pathway in which trauma-related emotional processing deficits increase vulnerability to NSSI through maladaptive coping behaviors.

Although prior research has examined the association between childhood trauma and NSSI among adolescents with depressive disorders, most studies have focused predominantly on emotional dysregulation as a mediating mechanism. However, the combined or sequential mediating effects of alexithymia and coping strategies remain underexplored. It is unclear whether these two variables act independently or form a chain mediation pathway in explaining the link between early trauma and NSSI. Therefore, the present study aimed to investigate the mediating roles of alexithymia and coping strategies in the relationship between childhood trauma and NSSI among adolescents with depressive disorders. We hypothesized that: (1) childhood trauma would positively predict NSSI; (2) alexithymia would mediate the relationship between childhood trauma and NSSI; (3) coping strategies would mediate the relationship between childhood trauma and NSSI; and (4) alexithymia and coping strategies would form a sequential (chain) mediation model linking childhood trauma and NSSI.

## 2 Methods

### 2.1 Participants and research design

From January 2024 to April 2024, adolescents with depressive disorders accompanied by NSSI behavior who met the inclusion criteria were recruited from the inpatient and outpatient departments of the hospital. Because participants were recruited from a psychiatric hospital, the sample may represent adolescents with more severe psychopathology, which may limit the generalizability of the findings to community populations. Diagnoses and assessments were independently confirmed by two qualified psychiatrists. Subjects meeting the following criteria were included in the study: A. Outpatients or inpatients who meet the DSM-5 diagnostic criteria for ‘depressive episode’ and are able to understand and independently complete the questionnaire content; B. Meets the DSM-5 diagnostic criteria for ‘non-suicidal self-injury’; C. Aged 12–18 years; D. Informed consent has been obtained from the patient and their legal guardian. The following patients were excluded: those with severe physical illnesses or substance abuse of alcohol and other psychoactive substances, those with comorbid severe mental disorders (such as bipolar disorder, schizophrenia, etc.), intellectual disability, or organic mental disorders. Subsequently, face-to-face interviews and questionnaire surveys were conducted with the subjects. A total of 138 questionnaires were distributed, of which 129 were valid, resulting in an efficiency rate of 93.5%. In addition, the relatively small sample size and the predominance of female participants should be considered when interpreting the results. Due to the cross-sectional design, causal relationships cannot be inferred.

The study was approved by the Ethics Committee of Guangdong Provincial People’s Hospital (Approval No.KY2024-1007-04). Written informed consent was obtained from all participants and their legal guardians prior to participation, in accordance with the Declaration of Helsinki.

### 2.2 Research tools

#### 2.2.1 General survey questionnaire

Demographic information was collected using a self-designed questionnaire, specifically including: gender (male, female), age, ethnicity (Han, other), and educational level (elementary, junior high, high school or technical secondary school, university).

#### 2.2.2 Childhood trauma questionnaire

This study used the Chinese version of the CTQ-SF questionnaire, compiled by Zhao Xing fu (16), to assess the level of childhood trauma in participants. The questionnaire contains a total of 28 items, with 3 validity items (10, 16, 22) not included in the total score. The five dimensions are emotional abuse, emotional neglect, sexual abuse, physical abuse, and physical neglect. The questionnaire uses a 5-point Likert scale, with ‘1=Never’ to ‘5=Always.’ Higher scores indicate more severe trauma experiences. The criteria for trauma assessment in this study are as follows: Emotional neglect ≥10 points, physical neglect ≥9 points, emotional abuse ≥9 points, physical abuse ≥8 points, sexual abuse ≥6 points. Scores exceeding the critical value indicate that the individual has experienced that type of trauma (17). The internal consistency of the CTQ-SF was good in the current sample(α=0.797).

#### 2.2.3 Adolescent non-suicidal self-injury assessment questionnaire

This study employed the self-injury behavior sub-questionnaire from the Adolescent Non-Suicidal Self-Injury Behavior Assessment Questionnaire developed by Wan Yuhui (18) to assess the frequency of NSSI behavior in participants over the past year. The self-injury behavior questionnaire contains a total of 12 items, divided into organized and disorganized self-injury behaviors, with scores ranging from 0 (never) to 4 (always). Higher scores indicate more frequent NSSI behavior. The internal consistency of the ANSAQ was good in the current sample(α=0.824).

#### 2.2.4 Chinese version of the Toronto alexithymia scale (TAS-20-C)

We used the Chinese version of the TAS-20 scale developed by Yi Jinyao (19), which includes a total of 20 items divided into three dimensions: difficulty identifying feelings, difficulty describing feelings, and externally oriented thinking. The scale uses a 5-point Likert scoring method, ranging from ‘strongly disagree’ to ‘strongly agree,’ scored 1–5 respectively, with higher scores indicating a more severe degree of alexithymia. Items 4, 5, 10, 18, and 19 are scored in reverse. The internal consistency of the TAS-20-C was good in the current sample(α=0.778).

#### 2.2.5 Simplified coping style questionnaire

This study utilizes the Simplified Coping Styles Questionnaire revised and compiled by Jie Yanning (20). The questionnaire contains a total of 20 items, including two dimensions: positive coping and negative coping. The scale uses a four-point Likert scoring system, ranging from ‘not at all’ to ‘often,’ scored from 0 to 3 points. The average score of each dimension reflects the type of coping strategy tendency adopted by the individual. In this study, the total scores of positive coping strategies and negative coping strategies will be analyzed separately. The SCSQ questionnaire has good reliability and validity among adolescent groups in China (21). The overall α coefficient of the coping strategies questionnaire (SCSQ) used in this study is 0.696, indicating good reliability of the scale. Among them, the internal consistency of the positive coping strategies subscale was good in the current sample(α=0.775), which is relatively low but still within an acceptable range for the negative coping strategies subscale(α=0.606). This may be because the sample size in this study is small and applied to a clinical sample. Therefore, results involving this subscale should be interpreted with caution.

### 2.3 Data analysis

The results of the paper-based survey questionnaire were entered into an Excel database. After excluding invalid questionnaires, the data were imported into SPSS 23.0 software. A common method bias test was conducted using Harman’s single-factor test. No serious common method bias was detected (the first factor explained 15.89% of variance, below the 40% threshold). Descriptive statistical analyses were then performed. A χ² test was used to compare differences in childhood trauma across demographic groups. Pearson correlation analysis was conducted, and a mediation model was constructed using the PROCESS 4.0 plugin developed by Hayes. Specifically, chain mediation analysis was performed with Model 6 to examine the mediating role of alexithymia, positive coping strategies, and negative coping strategies in the relationship between childhood trauma and NSSI among adolescents with depressive disorders. The mediating effect sizes of each pathway were estimated using the Bootstrap method with 5,000 resamples to calculate the 95% confidence intervals. Effects were considered statistically significant if the confidence interval did not include zero.

## 3 Results

### 3.1 Sample characteristics and childhood trauma subtypes (N = 129)

Among the 129 adolescents with depressive disorders and NSSI behaviors, the majority were female (92.2%), and most were enrolled in middle school (60.5%) or high school/technical secondary school (36.4%). The mean age was 14.74 years (SD = 1.59, range = 12–18), and nearly all participants were Han Chinese (96.9%). With respect to childhood trauma, emotional neglect (96.1%) and emotional abuse (77.5%) were the most prevalent subtypes, followed by physical neglect (67.4%) and physical abuse (35.7%). Sexual abuse was reported by 20.1% of participants. These findings highlight that emotional neglect and emotional abuse were the predominant adverse experiences in this clinical sample. The detailed demographic and trauma characteristics are presented in **Table 1**.

**Table 1:** Sociodemographic Characteristics of Adolescents with Depressive Disorders and NSSI Behavior (N = 129).

Project	Category	Frequency	Percentage (%)
Gender	Male	10	7.8
	Female	119	92.2
Education Level	Primary School	4	3.1
	Middle School	78	60.5
	High school (or technical secondary school, technical technology School)	47	36.4
	University	0	0
Ethnicity	Han Chinese	125	96.9
	other	4	3.1
Age(years)	Mean (SD) = 14.74 (1.59), Range = 12–18		
Childhood Trauma Subtypes	Emotional Neglect	124	96.1
	Emotional Abuse	100	77.5
	Physical Neglect	87	67.4
	Physical Abuse	46	35.7
	Sexual Abuse	27	20.1

### 3.2 Psychosocial factors score and clinical cut-offs (N = 129)

The median total childhood trauma score was 54 [43, 60], which exceeded the clinical cut-off (≥36), indicating high levels of adverse childhood experiences in this sample. Subscale analysis revealed that both emotional neglect (median = 18, cut-off ≥10) and emotional abuse (median = 12, cut-off ≥9) were markedly above established thresholds. The median NSSI frequency score was 16 [11, 24], reflecting moderate to high levels of self-injurious behaviors. For alexithymia, the total score was 72 [66, 77], also exceeding the clinical cut-off (≥61), suggesting notable emotional processing difficulties. Negative coping strategies showed relatively high scores (median = 17 [15, 23]), whereas results for other sub-dimensions are reported in the Supplementary Material. A full summary of psychosocial factor scores is provided in **Table 2**.

**Table 2:** Key Psychosocial Factor Scores and Clinical Cut-offs (N = 129).

Variable	Median [P25, P75]	SD	Clinical Cut-off	Interpretation
Total Childhood Trauma Score	54 [43, 60]	11.81	≥ 36 (any trauma)	Above cut-off
Emotional Neglect	18 [15, 21]	4.29	≥ 10	Above cut-off
Emotional Abuse	12 [9, 16]	4.63	≥ 9	Above cut-off
NSSI Frequency	16 [11, 24]	8.65	–	Moderate to high
Alexithymia Total Score	72 [66, 77]	8.73	≥ 61	Above cut-off
Negative Coping	17 [15, 23]	5.67	–	Higher tendency

Other sub-dimensions (e.g., physical neglect, physical abuse, sexual abuse, TAS-20 subscales, positive coping) are reported.

### 3.3 Correlations among childhood trauma, NSSI, alexithymia, and coping strategies (N = 129)

Using Pearson correlation analysis, we analyzed the correlation between participants’ NSSI behaviors and childhood trauma, alexithymia, positive coping strategies, negative coping strategies, as well as gender, age, ethnicity, and educational level. The correlation matrix is presented in **Table 3**.

**Table 3:** Correlations among childhood trauma, NSSI, alexithymia, and coping strategies (N = 129).

Variable	Childhood Trauma	NSSI	Alexithymia	Negative Coping	Positive Coping
Childhood Trauma	–	0.599***	0.525***	0.338***	–0.392***
NSSI	0.599***	–	0.515***	0.460***	–0.293**
Alexithymia	0.525***	0.515***	–	0.411***	–0.370***
Negative Coping	0.338***	0.460***	0.411***	–	0.035 (ns)
Positive Coping	–0.392***	–0.293**	–0.370***	0.035 (ns)	–

**p < 0.01, ***p < 0.001; NSSI, non-suicidal self-injury; ns, non-significant.

Correlation analyses showed that childhood trauma was significantly positively correlated with NSSI (r = 0.599, p < 0.001), alexithymia (r = 0.525, p < 0.001), and negative coping strategies (r = 0.338, p < 0.001), while significantly negatively correlated with positive coping strategies (r = –0.392, p < 0.001). Similarly, NSSI was positively associated with alexithymia (r = 0.515, p < 0.001) and negative coping strategies (r = 0.460, p < 0.001), and negatively associated with positive coping strategies (r = –0.293, p < 0.01). In addition, alexithymia was positively correlated with negative coping strategies (r = 0.411, p < 0.001) and negatively correlated with positive coping strategies (r = –0.370, p < 0.001). No significant correlation was found between negative and positive coping strategies.

### 3.4 Mediation and chain mediation analysis

Mediation analyses revealed that childhood trauma significantly predicted NSSI both directly and indirectly. The direct effect accounted for 69.25% of the total effect (β = 0.304, 95% CI: 0.186–0.422, p < 0.001). Detailed path coefficients and indirect effects are presented in **Table 4**.

Notably, the indirect effect through alexithymia accounted for the largest proportion of the total effect (β = 0.075, 95% CI: 0.086–0.149, p < 0.01), accounting for 17.08% of the total effect, which indicating its central role in the mediation model. Negative coping strategies also played a mediating role (β = 0.032, 95% CI: 0.002–0.084, p < 0.05), contributing 7.29% of the total effect. In addition, alexithymia exerted an indirect effect through its impact on negative coping, forming a significant chain mediation pathway (β = 0.028, 95% CI: 0.004–0.058, p < 0.05), accounting for 6.38% of the total effect.

In contrast, the indirect pathway involving positive coping was non-significant (β = –0.020, 95% CI: –0.021–0.073, p > 0.05), indicating that positive coping did not mediate the link between childhood trauma and NSSI.

**Figure 1:**
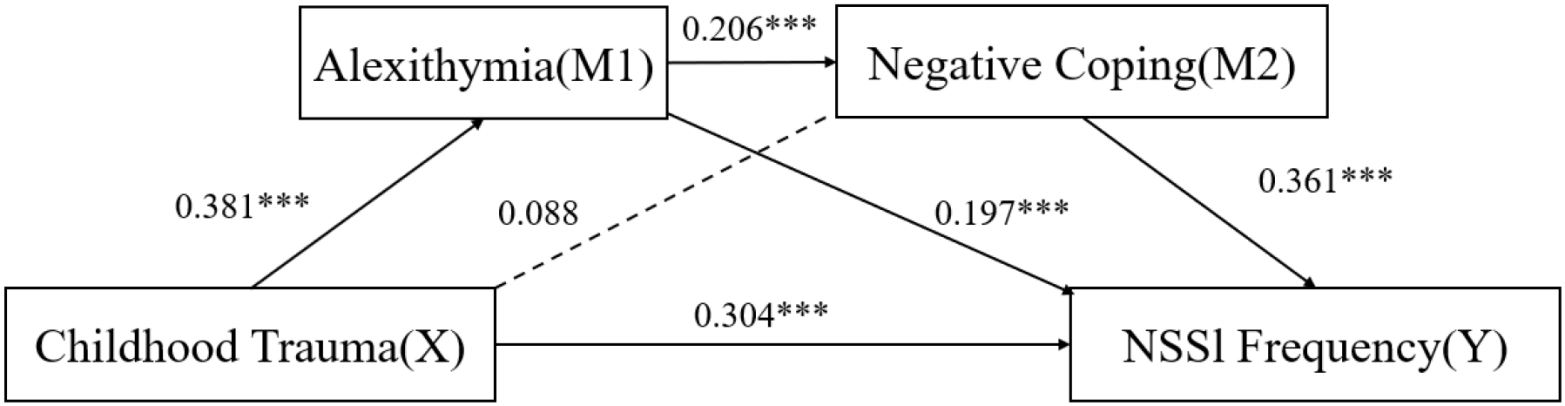
Mediation and chain mediation model of alexithymia and coping strategies linking childhood trauma and NSSI (N = 129). Solid lines indicate significant paths (p < 0.05), dashed lines indicate nonsignificant paths; Path coefficients in figure represent standardized regression weights within the model, corresponding to indirect effect estimates summarized in **Table 4**.

Overall, the total effect of childhood trauma on NSSI was 0.439 (95% CI: 0.331–0.547, p < 0.001), with 30.75% explained by indirect pathways. The integrated mediation model is presented in **Figure 1**, where significant paths are shown with solid arrows and non-significant paths with dashed arrows. Standardized path coefficients (β) are displayed to illustrate the relative strength of each association within the model. Bootstrapped 95% confidence intervals (5,000 resamples) confirmed that all indirect paths reported in **Table 4** were significant, as shown in **Figure 1**.

**Table 4:** Mediation and chain mediation effects of alexithymia and coping strategies in the association between childhood trauma and NSSI (N = 129).

Pathway	Effect size (β)	95% CI	Proportion of total effect	Significance
Direct effect	0.304	(0.186, 0.422)	69.25%	***
Indirect effect 1: Childhood trauma → Alexithymia → NSSI	0.075	(0.0086, 0.149)	17.08%	**
Indirect effect 2: Childhood trauma → Negative coping → NSSI	0.032	(0.002, 0.084)	7.29%	*
Indirect effect 3 (chain): Childhood trauma → Alexithymia → Negative coping → NSSI	0.028	(0.004, 0.058)	6.38%	*
Positive coping (non-significant)	–0.020	(–0.021, 0.073)	–	ns
Total effect	0.439	(0.331, 0.547)	100%	***

*p < 0.05, **p < 0.01, ***p < 0.001; ns, non-significant. NSSI, non-suicidal self-injury.

## 4 Discussion

This study examined the mediating roles of alexithymia and coping strategies in the relationship between childhood trauma and NSSI among adolescents with depressive disorders. The findings revealed that childhood trauma was positively associated with NSSI, and that both alexithymia and negative coping strategies partially mediated this association, forming a significant chain mediation pathway. In contrast, positive coping strategies did not exert a significant mediating effect. These results highlight the complex interplay between early adverse experiences, emotional processing deficits, and maladaptive coping in the development of NSSI. Notably, this study extends previous research by integrating alexithymia and coping styles within a single model, providing a more comprehensive explanation of how childhood trauma contributes to self-injurious behaviors in clinically depressed adolescents.

These findings are largely consistent with previous studies showing that childhood trauma—particularly emotional neglect and abuse—is a robust predictor of NSSI among adolescents with depression (22, 23). The mediating role of alexithymia and negative coping identified in our study also aligns with earlier evidence linking emotional processing deficits and maladaptive coping to self-injurious behaviors (24, 25). However, unlike several community-based studies reporting a protective effect of positive coping (26, 27), our results showed that positive coping did not significantly mediate the trauma–NSSI relationship, likely due to the clinical severity and reduced coping flexibility of this inpatient sample. In addiction, deficits in emotional awareness constrain the effective use of adaptive coping strategies, even when such strategies are theoretically available. Extending prior work, the current study integrated alexithymia and coping into a unified chain mediation model, demonstrating that childhood trauma may increase NSSI risk through emotion-processing deficits that foster maladaptive coping patterns, thereby offering a more comprehensive psychological mechanism consistent with contemporary models of self-injury (7, 28).

The observed mediation pathway can be interpreted through the lens of both emotional processing and coping theories. Early traumatic experiences may disrupt the development of emotional awareness and regulation, leading to difficulties in identifying and describing emotions—a core feature of alexithymia. This emotional processing deficit, in turn, limits the individual’s capacity to manage distress adaptively, increasing the likelihood of adopting maladaptive coping strategies such as avoidance, withdrawal, or self-blame (29, 30). These maladaptive strategies may provide short-term relief from overwhelming affect but ultimately reinforce NSSI through negative reinforcement mechanisms. From a theoretical perspective, this chain mediation model accords with Nock’s four-function model (7), which conceptualizes NSSI as serving intrapersonal emotion-regulation and interpersonal signaling functions. It also supports the experiential avoidance model (28), suggesting that NSSI is maintained by attempts to escape intolerable emotional states. Together, these findings delineate a coherent psychological mechanism in which childhood trauma fosters alexithymia and maladaptive coping, thereby heightening vulnerability to self-injury among adolescents with depression.

These findings have important clinical and public health implications. Clinically, they underscore the necessity of assessing childhood trauma histories and emotional processing difficulties in adolescents presenting with depressive symptoms and self-injurious behaviors. Targeted interventions focusing on emotion identification, expression, and regulation—such as emotion-focused therapy or mindfulness-based cognitive therapy—may help reduce alexithymia and maladaptive coping tendencies, thereby decreasing NSSI risk (28). Moreover, strengthening adaptive coping and problem-solving skills should be incorporated into psychotherapeutic programs for this population. From a broader perspective, early identification of trauma exposure and alexithymic traits in school and community settings may allow for timely prevention and psychoeducation. Public health strategies aimed at promoting trauma-informed care, enhancing emotional literacy, and reducing stigma associated with self-injury could play a crucial role in mitigating the long-term psychological consequences of childhood adversity.

## 5 Limitations

Several limitations of this study should be acknowledged. First, the relatively small sample size and single-center recruitment limit the generalizability of the findings. Future research should replicate these results in larger, more diverse, and multicenter samples to enhance external validity. Second, the gender imbalance in our sample (92.2% female) may have influenced the observed associations, as previous studies suggest gender differences in both alexithymia and NSSI prevalence (17). Third, the cross-sectional design precludes causal inference; longitudinal studies are needed to clarify the temporal sequence of childhood trauma, alexithymia, coping strategies, and NSSI. Finally, although self-report measures are widely used in psychological research, they may be subject to recall and reporting biases. Future studies should integrate multi-informant data or experimental paradigms to improve measurement accuracy. Despite these limitations, the present study provides novel insights into the mechanisms linking early trauma and self-injury in adolescents with depression and highlights several promising avenues for future clinical and empirical work.

## 6 Conclusion

This study provides evidence that childhood trauma is significantly associated with NSSI among adolescents with depressive disorders. This association was partially mediated by alexithymia and maladaptive coping strategies, and a sequential mediation pathway through alexithymia and negative coping was supported. These findings underscore the importance of assessing trauma-related emotional processing deficits and coping patterns in the clinical evaluation and intervention of adolescents engaging in NSSI. Future research should incorporate longitudinal designs and multi-center samples to explore other potential mediating factors, such as self-criticism, social support, and biological indicators, to offer a more comprehensive theoretical and empirical basis for preventing and intervening in NSSI behaviors.

## Data Availability

The raw data supporting the conclusions of this article will be made available by the authors, without undue reservation.
